# Spinal motor neuron degeneration after brachial plexus avulsion: mechanisms and therapeutic targets

**DOI:** 10.3389/fnins.2026.1778229

**Published:** 2026-02-16

**Authors:** Jiantao Yang, Bengang Qin, Yixi Yang, Jintao Fang, Wenting He

**Affiliations:** Department of Microsurgery, Orthopedic Trauma and Hand Surgery, The First Affiliated Hospital, Sun Yat-sen University, Guangzhou, China

**Keywords:** brachial plexus avulsion, ferroptosis, neuroinflammation, neuroprotection, programmed cell death, pyroptosis, regenerative therapy, spinal motor neuron

## Abstract

Brachial plexus avulsion injury (BPAI) causes violent detachment of cervical ventral roots and rapid degeneration of spinal motor neurons (SMNs), driven by abrupt energy failure, axonal disconnection, and a rapidly escalating neuroinflammatory microenvironment. Current evidence demonstrates that SMN loss arises from the coordinated activation of multiple programmed cell death (PCD) pathways-including apoptosis, necroptosis, pyroptosis, ferroptosis, and impaired autophagy-regulated by oxidative stress, mitochondrial dysfunction, calcium overload, iron dyshomeostasis, and inflammasome activation. Crosstalk among these pathways, amplified by microglial priming, macrophage infiltration, and astrocytic reactivity, forms a self-propagating neurodegenerative network extending beyond the avulsion site. This review integrates recent mechanistic advances, highlighting key regulators such as the METTL14-EEF1A2 m^6^A axis, RIPK1/RIPK3/MLKL necrosome, NLRP3-GSDMD pyroptotic signaling, GPX4-dependent ferroptosis, and AR-SIRT1-AMPK-mTOR-mediated autophagy disruption. We further summarize emerging interventions-including PCD-targeted inhibitors, immunomodulatory therapies, biomaterial-based delivery systems, gene therapy, and neural organoid transplantation-toward precision neuroprotection and improved functional recovery after BPAI.

## 1 Introduction

Brachial plexus avulsion injury (BPAI) is among the most devastating forms of peripheral nerve trauma, caused by high-energy longitudinal traction forces that detach cervical ventral roots from the spinal cord. In contrast to peripheral nerve transection or stretch injuries, BPAI directly disrupts central motor circuitry within the spinal gray matter, triggering a rapid and progressive degeneration of spinal motor neurons (SMNs) that cannot be fully reversed by current surgical interventions (Carlstedt, 2008; Boyle et al., 2025). Although root reimplantation can partially restore anatomical continuity between the spinal cord and peripheral nerves, long-term functional recovery remains limited. This poor outcome is largely attributable to the early and substantial loss of SMNs, which occurs predominantly within the first weeks following avulsion and critically constrains the regenerative capacity of the motor system (Cai et al., 2021; Vancea et al., 2025).

The pathophysiology of BPAI reflects a dynamic interplay among mechanical disruption, metabolic collapse, and progressive neuroinflammatory remodeling. Ventral root avulsion abruptly interrupts retrograde axonal transport, depriving SMNs of essential trophic support and destabilizing mitochondrial homeostasis (Li et al., 2022). Within hours of injury, oxidative stress, calcium overload, and early microglial activation converge to initiate multiple PCD pathways. Increasing evidence suggests that SMNs degeneration after BPAI cannot be explained by a single death mechanism; instead, apoptosis, necroptosis, pyroptosis, ferroptosis, and impaired autophagy are activated in parallel and interact synergistically to accelerate neuronal loss (Shi et al., 2021; Song et al., 2024). These processes are further shaped by a progressively dysregulated immune-glia–neuron microenvironment, in which reactive astrocytes, infiltrating macrophages, and activated microglia amplify neuronal vulnerability through sustained cytokine signaling, redox imbalance, and inflammasome activation (Avraham et al., 2021; Zhong et al., 2021).

Recent studies have expanded the mechanistic landscape by identifying epigenetic and post-transcriptional regulators, including m^6^A RNA methylation, microRNAs, and chromatin remodeling, which modulate SMN stress responses and cell death thresholds after BPAI (Ni et al., 2022; Cheng et al., 2023; Zeng et al., 2023). Parallel advancements in single-cell transcriptomics and spatial transcriptomics have provided a high-resolution understanding of injury propagation within spinal circuits, revealing region-specific vulnerabilities and distinct subpopulations of injury-responsive microglia and astrocytes (Sathyamurthy et al., 2018; Gao et al., 2023; Hu and Gao, 2025).

The clinical implications of these mechanistic insights are substantial. A new generation of targeted intervention strategies has emerged, including selective inhibitors of PCD pathways (e.g., RIPK1 inhibitors, NLRP3 inhibitors, ferroptosis blockers), biomaterial-based delivery systems for neurotrophic factors, and regenerative therapies based on induced pluripotent stem cell-derived motor neuron organoids (Huang Y. et al., 2022; Lopes et al., 2022; Huang et al., 2023; Liao et al., 2024). These strategies offer promising avenues to preserve SMN viability, optimize the microenvironment for axonal regeneration, and enhance the outcomes of surgical root reimplantation.

This review integrates current advances in our understanding of SMN death mechanisms after BPAI and evaluates emerging therapeutic strategies from molecular, cellular, and translational perspectives. By consolidating evidence from basic neuroscience, immunobiology, and regenerative medicine, we aim to provide a comprehensive framework for guiding future research and improving clinical management of BPAI-induced neurodegeneration.

## 2 Neuropathological responses following BPAI

BPAI initiates a complex cascade of neuropathological events characterized by a dynamic and ultimately maladaptive imbalance. Although injured neurons transiently activate intrinsic regenerative programs, the extreme severity of root avulsion simultaneously engages powerful degenerative mechanisms that progressively overwhelm reparative capacity, culminating in irreversible neuronal loss.

### 2.1 Neuronal soma responses

The neuronal soma represents a critical decision point between regeneration and degeneration following BPAI. Immediately after injury, affected neurons-including SMNs, dorsal root ganglion (DRG) sensory neurons, and sympathetic neurons-undergo rapid morphological and metabolic remodeling known as chromatolysis (He and Jin, 2016).

Histologically, chromatolysis is marked by somatic swelling, nuclear eccentricity, and dispersion of Nissl substance. Rather than a purely degenerative change, chromatolysis represents an active adaptive response that supports neuronal survival and axonal regeneration. Following injury, neurons undergo marked metabolic reprogramming, with RNA synthesis increasing by 300 ~ 500% and the rough endoplasmic reticulum reorganizing into dispersed ribosomal granules to enhance translational capacity. Within a critical window of approximately 4–20 days post-injury, neurons display a robust biosynthetic surge, characterized by increased rRNA/tRNA ratios and ribosome assembly into “protein synthesis factories,” enabling efficient production of cytoskeletal proteins such as tubulin and neurofilaments required for axonal growth and transport (Lieberman, 1971; Fawcett and Verhaagen, 2018).

However, the distinctive mechanical severity of BPAI-caused by violent traction forces that forcibly detach ventral roots from the spinal corddrives neurons toward a degenerative tipping point. Abrupt interruption of retrograde neurotrophic signaling (e.g., NGF, BDNF) initiates a cascade of cellular dysfunction marked by mitochondrial failure, ATP depletion, calcium overload, and activation of both caspase-dependent apoptotic pathways and RIPK1-MLKL-mediated necroptosis (Fricker et al., 2018). As these degenerative signals intensify, they irreversibly shift neurons from a regenerative trajectory toward retrograde degeneration, ultimately leading to soma death and permanent loss of regenerative potential. Disruption of the delicate balance between anabolic repair and catabolic degeneration thus represents a defining event in the pathophysiological progression of BPAI.

### 2.2 Axonal regeneration

Axonal regeneration is a tightly coordinated molecular process governed by the spatiotemporal regulation of immediate-early gene (IEG) expression. These genes function as molecular switches that determine whether injured neurons successfully engage in regenerative programs or instead enter maladaptive states that promote degeneration.

#### 2.2.1 Regeneration initiation phase

Axonal regeneration is initiated by a rapid injury-induced rise in intracellular Ca^2+^ (1–10 μM), which activates Ca^2+^/calmodulin-dependent protein kinase II (CaMKII). Activated CaMKII drives phosphorylation of cAMP response element-binding protein (CREB), forming a core CaMKII-CREB axis that triggers transcription of regeneration-associated genes (Ghosh and Greenberg, 1995; Wayman et al., 2008). This signaling cascade promotes regeneration through coordinated chromatin remodeling and IEG induction. Recruitment of transcriptional coactivators such as CBP/p300 enhances histone acetylation and transcriptional accessibility, while rapid upregulation of IEGs including c-FOS and JUN family members occurs within 0.5–2 h post-injury, establishing the transcriptional program for axonal growth (Herdegen and Leah, 1998; Riccio, 2010). Importantly, the magnitude of Ca^2+^ signaling encodes injury severity: root avulsion elicits stronger Ca^2+^ transients and markedly higher c-FOS induction than distal nerve injury, thereby defining an intensity-dependent transcriptional threshold for regenerative activation.

#### 2.2.2 Regeneration maintenance phase

During the regeneration maintenance phase, neurotrophic signaling establishes negative feedback circuits that stabilize early injury-induced transcription while preventing metabolic overload. Retrogradely transported neurotrophins, particularly NT-3 and BDNF, activate Trk-dependent Ras-MAPK signaling, which induces transcriptional corepressors such as NAB1/2 to dampen excessive IEG activity (Harrington and Ginty, 2013). Concurrently, recruitment of histone deacetylases (HDACs) to IEG promoters promote chromatin condensation and limits sustained transcriptional elongation (O'Donnell and Meaney, 2020).

In parallel, phosphorylation-dependent relocalization of Tau modulates its nuclear availability, interfering with CREB-coactivator interactions and further restraining injury-driven transcription. These feedback mechanisms constrain mTORC1-dependent protein synthesis to preserve cellular ATP levels and reduce oxidative stress by limiting excessive oxygen species (ROS) accumulation (Sultan et al., 2011). Together, these coordinated transcriptional, epigenetic, and metabolic controls establish a transient homeostatic state that supports continued axonal elongation while protecting neurons from energetic failure during brachial plexus avulsion-induced regeneration.

## 3 Distal axons

According to the classical Seddon classification, BPAI corresponds to the most severe category of peripheral nerve injury-neurotmesis-defined by complete disruption of the axon together with all surrounding connective tissue components, including the endoneurium. This extreme structural disintegration initiates a cascade of profound and largely irreversible degenerative processes within the distal axonal compartment, fundamentally compromising axonal integrity and long-term regenerative potential.

### 3.1 Wallerian degeneration

Within 72 h after injury, the distal axonal segment undergoes classical Wallerian degeneration, marked by rapid axonal fragmentation, cytoskeletal collapse, and disintegration of axonal structure. Concurrently, Schwann cells rapidly transition to a repair phenotype, characterized by dedifferentiation, enhanced phagocytic activity, and efficient clearance of axonal and myelin debris. This coordinated response transiently creates a growth-permissive microenvironment that normally supports axonal regeneration. However, in brachial plexus avulsion, regeneration from the proximal compartment is largely unsuccessful due to irreversible loss of axonal continuity at the nerve root and progressive spinal motor neuron degeneration, ultimately preventing effective reinnervation (Jessen and Mirsky, 2016; Coleman and Höke, 2020).

### 3.2 Target organ denervation

After distal axonal degeneration, loss of neural input drives target tissues-particularly skeletal muscle and skin-into chronic denervation. In skeletal muscle, sustained denervation leads to progressive myofiber atrophy, fibrotic infiltration, and extensive extracellular matrix remodeling. At the neuromuscular junction (NMJ), deprivation of motor neuron-derived trophic and activity-dependent signals causes destabilization of acetylcholine receptor (AChR) clusters, followed by receptor dispersion, internalization, and postsynaptic structural collapse. When denervation persists for 12 ~ 18 months, these molecular and architectural alterations become largely irreversible, resulting in permanent NMJ dysfunction. Consequently, even delayed axonal reinnervation fails to restore effective neuromuscular transmission, severely limiting functional recovery (Tintignac et al., 2015; Li et al., 2018).

### 3.3 The “ghost synapse” trap and functional regeneration failure

Despite advances in microsurgical repair, functional recovery after BPAI remains primarily limited by early SMN loss. Clinical and experimental studies indicate that the first ~4 weeks post-injury constitute a critical prognostic window: when SMN survival falls below ~40%, regenerating axons that eventually reach target muscles predominantly form structurally immature and electrophysiologically silent contacts-termed “ghost synapses.” These pseudo-synaptic structures lack effective signal transmission, rendering anatomically reconstructed pathways functionally ineffectively. Together with Wallerian degeneration, irreversible target organ denervation, and early SMN depletion, ghost synapse formation represents a major biological barrier to meaningful functional recovery after BPAI (Gordon, 2020). These findings highlight the need for early, mechanism-driven neuroprotective interventions that preserve SMNs, shorten denervation duration, and promote formation of functional neuromuscular synapses rather than purely anatomical reconnection.

## 4 Cell death modes of SMNs and their regulatory mechanisms after BPAI

SMN loss after BPAI results from the coordinated activation and crosstalk of multiple regulated cell-death programs. This section outlines the molecular wiring of the major PCD pathways-apoptosis, necroptosis, pyroptosis, ferroptosis and autophagy as a modulatory process, and provides mechanistic insights and therapeutic entry points for each (Table 1).

**Table 1:** Regulated cell death modes of spinal motor neurons after BPAI.

Cell death mode	Core molecular triggers	Key signaling mediators	Cellular features	Therapeutic entry points
Apoptosis	Loss of retrograde trophic support; Mitochondrial stress; Oxidative injury	Bax/Bak, cytochrome c, caspase-9/-3; Fas/FasL; JNK, p53	Cell shrinkage; DNA fragmentation; Caspase activation	Caspase inhibitors; Akt/mTOR activation; Hsp27 induction; METTL14 inhibition
Necroptosis	Excess TNF-α; Caspase-8 inhibition; Inflammatory overload	RIPK1-RIPK3-MLKL	Membrane rupture; DAMP release; Strong inflammation	RIPK1 inhibitors (Necrostatin-1); TNF/TNFR blockade
Pyroptosis	ROS accumulation; Inflammasome activation	NLRP3, caspase-1, GSDMD, IL-1β, IL-18	Membrane pores; Cytokine release; Swelling	NLRP3 inhibitors; Caspase-1 inhibitors; Hv1 inhibition; NAG
Ferroptosis	Iron overload; Lipid peroxidation; GPX4 failure	Fe^2+^, ACSL4, GPX4, lipid ROS	Mitochondrial shrinkage; Membrane damage	Ferrostatin-1; Liproxstatin-1; Iron chelators; GPX4 delivery
Autophagy dysregulation	Energy depletion; ROS; AR upregulation	AMPK-ULK1-Beclin-1; mTOR; SIRT1	Autophagosome accumulation; Impaired flux	AR inhibition (epalrestat); Autophagic flux normalization

### 4.1 Molecular pathways governing SMN apoptosis after BPAI

After BPAI, irreversible SMN loss is predominantly driven by activation of convergent apoptotic pathways. Axonal disconnection abruptly interrupts retrograde neurotrophic signaling, destabilizes mitochondrial homeostasis, and induces oxidative stress, thereby engaging intrinsic apoptosis programs. In parallel, severe mechanical trauma and sustained neuroinflammation activate extrinsic death signaling, collectively lowering the apoptotic threshold of SMNs. Importantly, SMN apoptosis is not restricted to the acute phase but persists chronically: both clinical observations and animal studies demonstrate significantly higher apoptotic indices in proximal motor neurons months after injury, indicating a self-perpetuating degenerative process. This prolonged vulnerability highlights apoptosis as a central barrier to functional recovery and a critical target for early neuroprotective intervention.

#### 4.1.1 Stress-response genes and early apoptotic activation

Within hours after BPAI, severe mechanical trauma induces rapid intracellular Ca^2+^ overload and oxidative stress in SMNs. These early events activate stress-responsive transcription factors, most prominently c-Jun, which is consistently observed in injured motor neurons after root avulsion. Although transient c-Jun activation participates in injury signaling, sustained and excessive activation is closely associated with neuronal apoptosis rather than regeneration (Ham et al., 2000; Raivich and Behrens, 2006). Concomitantly, post-mitotic SMNs exhibit aberrant expression of cell cycle-related proteins, including cyclins and cyclin-dependent kinases. Such inappropriate cell cycle re-entry is a well-established mechanism of neuronal death in both central and peripheral nervous system injuries, leading to replication stress, DNA damage responses, and activation of apoptotic pathways (Becker and Bonni, 2004; Herrup and Yang, 2007).

**Figure 1:**
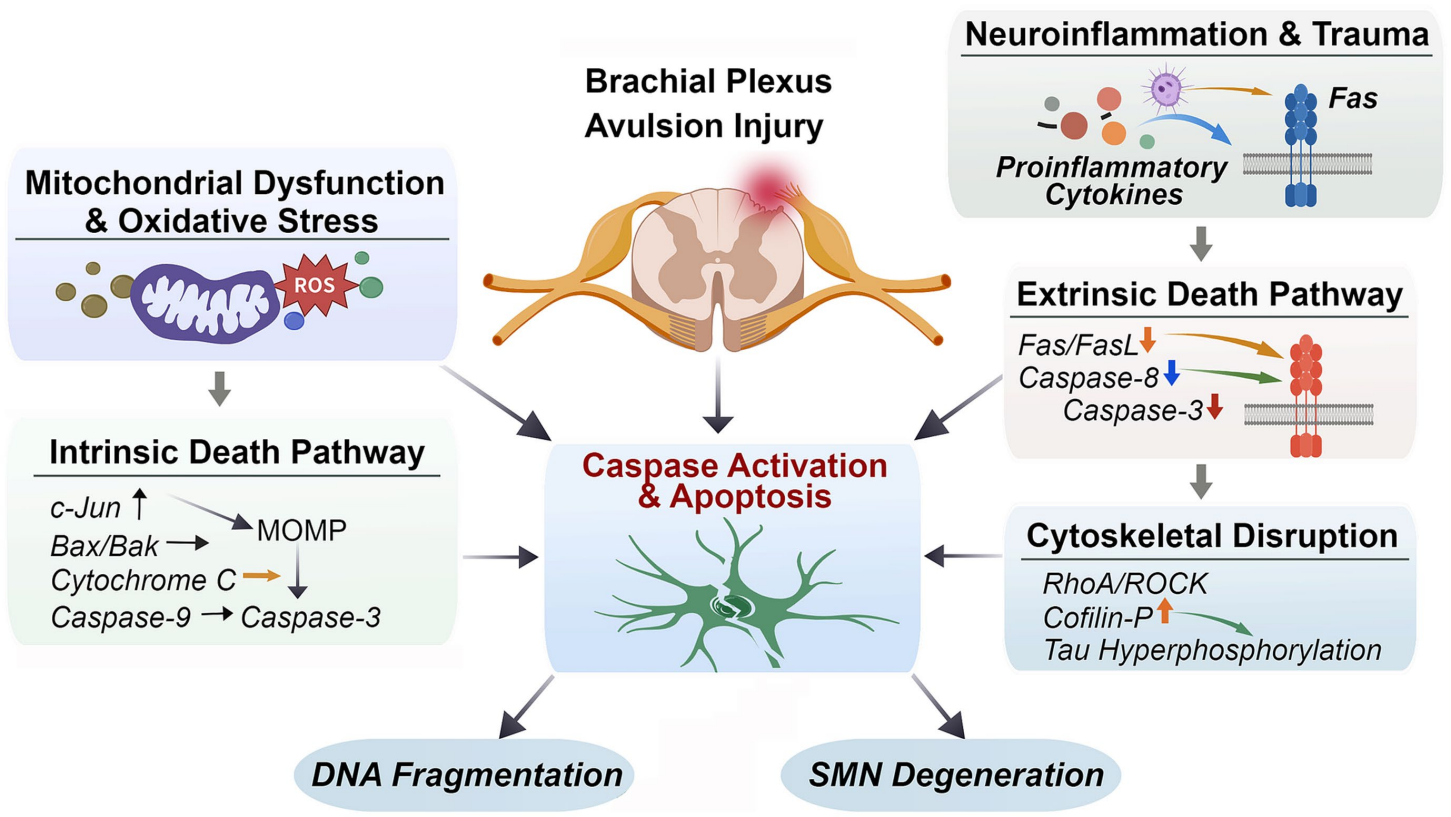
Molecular pathways governing SMN apoptosis after BPAI. This figure illustrates the convergent intrinsic and extrinsic apoptotic pathways driving SMN loss after BPAI. Axonal disconnection induces mitochondrial dysfunction and oxidative stress, activating intrinsic apoptosis via Bax/Bak-mediated mitochondrial outer membrane permeabilization, cytochrome *c* release, and caspase-9/3 activation. In parallel, neuroinflammation and trauma activate extrinsic death signaling through Fas/FasL and caspase-8. Cytoskeletal disruption mediated by RhoA/ROCK–cofilin and Tau hyperphosphorylation further amplifies apoptotic signaling. Together, these pathways culminate in caspase activation, DNA fragmentation, and progressive SMN degeneration after BPAI.

At the signaling level, pro-survival pathways such as PI3K/Akt are progressively suppressed, while stress-activated pathways including JNK and p53 are enhanced. This imbalance lowers the apoptotic threshold of SMNs (Xiao et al., 2022). Within 24–72 h after injury, mitochondrial dysfunction becomes evident, characterized by Bax/Bak-dependent mitochondrial outer membrane permeabilization, cytochrome c release, and subsequent activation of caspase-9 and caspase-3. In parallel, death receptor-mediated pathways, including Fas/FasL signaling, further amplify caspase activation (Krajewski et al., 1999; Eldadah and Faden, 2000). Together, these convergent mechanisms drive progressive and irreversible SMN apoptosis following BPAI (Figure 1).

#### 4.1.2 Heat shock protein 27: an endogenous neuroprotective shield

Endogenous stress-response pathways represent the first line of defense against injury-induced neuronal apoptosis. Among them, Heat Shock Protein 27 (Hsp27) functions as a molecular chaperone that stabilizes cytoskeletal elements, inhibits caspase activation, and maintains mitochondrial integrity. After BPAI, Hsp27 expression peaks in the ventral horn within 48–72 h post-injury, coinciding with the critical window for SMN survival (Wagstaff et al., 1999; Benn et al., 2002).

#### 4.1.3 The METTL14/EEF1A2/m^6^A epitranscriptomic axis

Recent studies indicate that m^6^A epitranscriptomic remodeling is a critical determinant of SMN survival after traumatic injury. Both BPAI-related models and spinal cord injury (SCI) are associated with a global increase in m^6^A RNA methylation, reflecting stress-induced reprogramming of post-transcriptional regulation (Wang et al., 2018; Dermentzaki and Lotti, 2020). Among m^6^A “writers,” METTL14 is consistently upregulated after SCI and promotes neuronal apoptosis. Mechanistically, METTL14-mediated m^6^A modification accelerates the degradation of specific pro-survival transcripts, including EEF1A2, a neuron-enriched translation elongation factor known to support Akt/mTOR signaling. Loss of EEF1A2 results in attenuation of Akt phosphorylation and reduced resistance to apoptotic stress (Wang et al., 2021). Importantly, genetic or pharmacological inhibition of METTL14 restores EEF1A2 expression, reactivates Akt/mTOR signaling, and significantly reduces neuronal apoptosis, accompanied by improved functional recovery in experimental SCI models (Saxton and Sabatini, 2017; Gao et al., 2022).

#### 4.1.4 Cytoskeletal regulatory pathways

Cytoskeletal integrity is essential for maintaining neuronal polarity, axonal transport, and survival signaling. After BPAI, mechanical disruption of actin-microtubule dynamics constitutes an early and central driver of SMN degeneration, linking structural instability to apoptotic activation.

Within hours of peripheral nerve injury, RhoA/ROCK signaling is rapidly activated, leading to LIMK1-dependent phosphorylation of cofilin. Phosphorylated cofilin suppresses actin turnover, induces growth cone collapse, and restricts axonal extension, while simultaneously facilitating pro-apoptotic signaling cascades. Pharmacological inhibition of ROCK (e.g., Fasudil or Y-27632) restores actin dynamics, preserves mitochondrial integrity, and significantly attenuates SMN apoptosis in experimental nerve and SCI models (Mueller et al., 2005; Wang et al., 2019).

In parallel, microtubule destabilization critically impairs axonal transport and mitochondrial distribution, resulting in energetic failure and progressive neuronal loss. Stabilization of microtubules using CNS-penetrant agents such as Epothilone B has been shown to reduce glial scarring, promote long-distance axonal regeneration, and improve functional recovery in preclinical SCI models, underscoring the therapeutic relevance of cytoskeletal preservation (Hellal et al., 2011; Ruschel et al., 2015; Duan et al., 2021). Dysregulation of microtubule-associated proteins provides an additional mechanistic link. Hyperphosphorylated Tau exhibits reduce microtubule-binding affinity, exacerbating cytoskeletal fragmentation and transport defects. In motor neurons, aberrant Tau signaling has been associated with impaired CREB-dependent transcription and heightened susceptibility to apoptosis (Millecamps and Julien, 2013).

Collectively, these findings define an integrated RhoA/ROCK-LIMK-cofilin-Tau signaling network that couples cytoskeletal disorganization to metabolic stress and apoptotic commitment (Figure 1). Targeting this network through ROCK inhibition or microtubule stabilization represents a promising strategy to simultaneously enhance neuronal survival and regenerative capacity after BPAI.

### 4.2 Programmed necrosis mechanisms and core signaling pathways

In addition to apoptosis, regulated necrosis (necroptosis) is a major contributor to SMN loss following BPAI, particularly during the early post-injury period. Necroptosis is a genetically controlled, pro-inflammatory form of cell death mediated by the RIPK1-RIPK3-MLKL signaling axis, and is preferentially activated when caspase-dependent apoptosis is impaired or overwhelmed (Vandenabeele et al., 2010).

**Figure 2:**
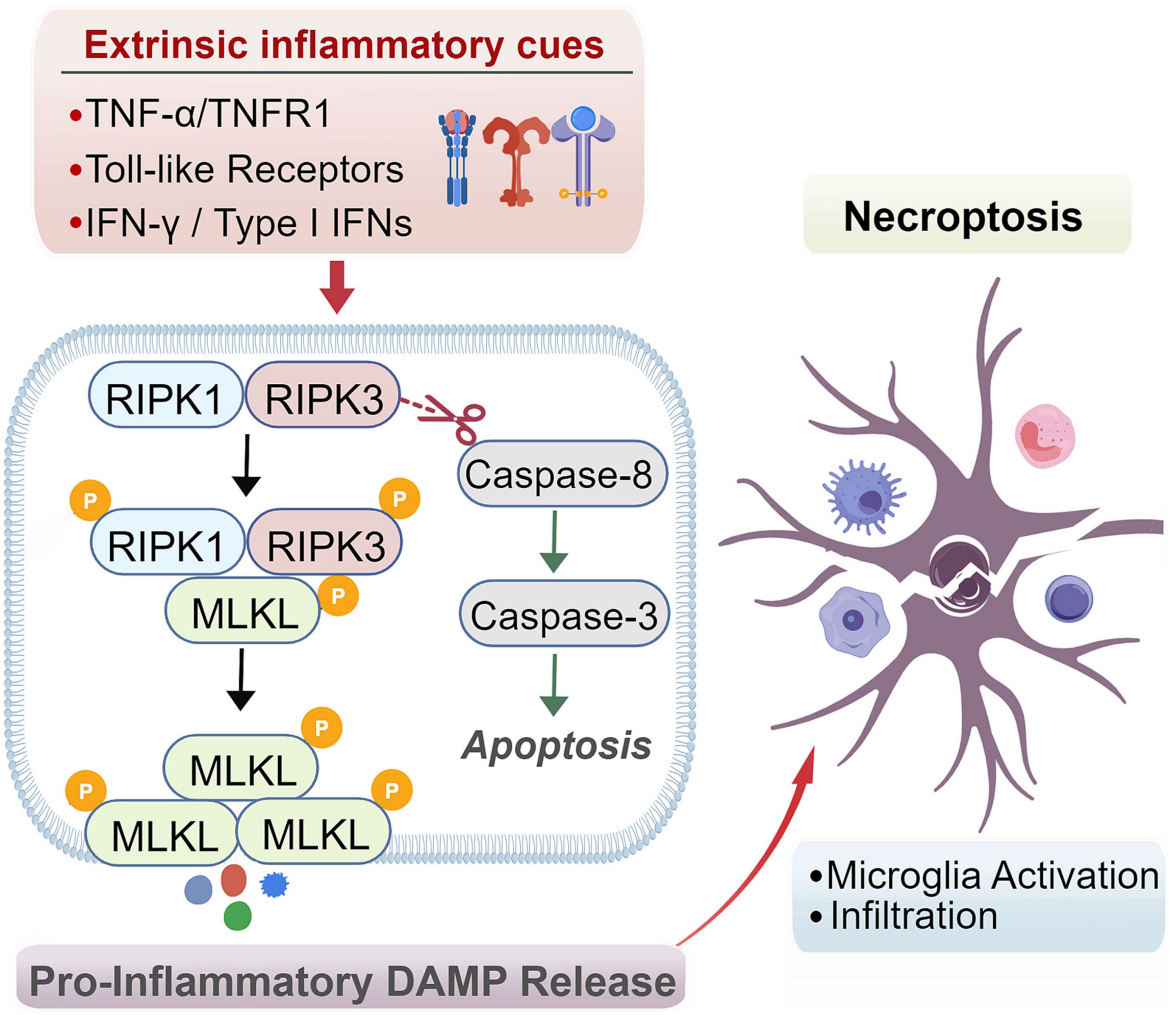
Necroptosis signaling in SMN loss after BPAI. This figure depicts the core necroptotic signaling pathways contributing to SMN degeneration following BPAI. Extrinsic inflammatory cues, including TNF-α/TNFR1, toll-like receptors, and interferon signaling, trigger RIPK1-RIPK3 necrosome formation and MLKL phosphorylation. Activated MLKL translocates to the plasma membrane, causing membrane permeabilization, ionic imbalance, and necrotic cell lysis. Caspase-8 functions as a critical checkpoint, suppressing necroptosis through cleavage of RIPK1 and RIPK3. Necroptotic membrane rupture releases pro-inflammatory DAMPs drive microglial activation and macrophage infiltration, thereby amplifying neuroinflammation and secondary neuronal loss.

Necroptosis is initiated by extrinsic inflammatory cues, including TNF-α/TNFR1 signaling, Toll-like receptor activation, and interferon pathways. Upon receptor engagement, RIPK1 associates with RIPK3 to form the necrosome, leading to phosphorylation and oligomerization of MLKL, which translocates to the plasma membrane and induces membrane permeabilization, ionic imbalance, and cell lysis. In SMNs after BPAI, necroptotic signaling emerges within hours to days after injury and temporally overlaps with apoptotic cascades, collectively accelerating early neuronal attrition (Ito et al., 2016; Yuan et al., 2019). A critical regulatory checkpoint between apoptosis and necroptosis is caspase-8. Active caspase-8 suppresses necroptosis by cleaving RIPK1 and RIPK3, whereas caspase-8 inhibition-resulting from severe cellular stress, mitochondrial dysfunction, or inflammatory signaling-unleashes necroptotic execution (Oberst et al., 2011). This crosstalk explains why apoptosis-targeted neuroprotection alone is often insufficient to prevent SMN loss after severe root avulsion (Figure 2).

Necroptotic SMN death is intrinsically pro-inflammatory. Membrane rupture leads to the release of damage-associated molecular patterns (DAMPs), such as HMGB1 and ATP, which activate microglia and infiltrating macrophages, establishing a feed-forward inflammatory loop that further amplifies neuronal loss and distal denervation. Consequently, necroptosis critically contributes to the rapid collapse of the motor neuron pool, defining a narrow therapeutic window for early intervention. Targeting necroptosis therefore represents a promising adjunct to apoptosis-focused strategies. RIPK1 inhibitors, such as Necrostatin-1 and its optimized derivatives, have demonstrated efficacy in reducing MLKL activation, limiting neuronal loss, and improving functional outcomes in experimental models of neural trauma (Degterev et al., 2005; Nishijima et al., 2023). Combined modulation of necroptosis, apoptosis, and neurotrophic signaling may offer a more effective, multimodal neuroprotective approach for preserving SMN viability after BPAI. The involvement of necroptotic signaling highlights the therapeutic potential of targeting RIPK1-RIPK3-MLKL-mediated pathways to limit inflammatory cell death and secondary tissue damage after BPAI.

### 4.3 Mechanisms and core signaling pathways of pyroptosis

Pyroptosis is a highly inflammatory form of PCD characterized by gasdermin-mediated plasma membrane pore formation, rapid ionic imbalance, and release of potent proinflammatory cytokines such as IL-1β and IL-18. Unlike apoptosis, pyroptotic neurons actively release DAMPs, thereby amplifying neuroinflammation and propagating secondary tissue injury (Shi et al., 2017). Increasing evidence indicates that pyroptosis contributes significantly to SMN loss following BPAI and SCI.

Pyroptotic signaling is primarily driven by inflammasome activation, most notably the NLRP3 inflammasome, which promotes caspase-1 activation and subsequent cleavage of gasdermin D (GSDMD). The N-terminal fragment of GSDMD oligomerizes to form membrane pores, leading to cell swelling, membrane rupture, and inflammatory mediator release (Broz and Dixit, 2016). Notably, neuronal pyroptosis often persists longer and extends beyond the immediate lesion site compared with apoptosis, indicating a sustained role in secondary neurodegeneration. Microglia act as critical upstream regulators of pyroptosis in the injured spinal cord. Excessive production of reactive ROS by activated microglia promotes NLRP3 inflammasome assembly. Genetic or pharmacological inhibition of microglial ROS generation-such as deletion of the voltage-gated proton channel Hv1-significantly attenuates NLRP3 activation, reduces neuronal pyroptosis, and preserves spinal cord tissue integrity (Al Mamun et al., 2021; Zheng et al., 2022). These findings position microglial ROS as a key mechanistic link between oxidative stress, neuroinflammation, and pyroptotic SMN injury.

Pyroptosis is not restricted to neurons but also occurs in peripheral glial cells. In injured peripheral nerves, Schwann cells undergo caspase-1-dependent pyroptosis, releasing inflammatory mediators that impair dorsal root ganglion neuron (DRGN) function. Pharmacological inhibition of pyroptosis in Schwann cells reduces local inflammation, enhances axonal regeneration, and improves functional recovery, highlighting the broader contribution of glial pyroptosis to nerve repair failure (Wang et al., 2023; Wang et al., 2024). Therapeutically, targeting pyroptotic signaling has emerged as a promising neuroprotective strategy. Inhibition of inflammasome components or gasdermin activation, as well as metabolic or anti-inflammatory interventions (e.g., N-acetylglucosamine), has been shown to reduce pyroptotic cell death, promote remyelination, and improve motor outcomes in experimental BPAI and SCI models. Collectively, these findings suggest that modulation of pyroptosis, particularly at the level of microglial inflammasome activation, may effectively limit secondary neuroinflammation and preserve SMN viability after severe nerve root injury (Amo-Aparicio et al., 2022; Wang et al., 2022; Broz, 2025). These findings implicate inflammasome-mediated pyroptosis as a potential therapeutic target, particularly through modulation of microglial activation and cytokine release to restrain inflammation-driven neuronal injury.

### 4.4 Ferroptosis pathways in proximal SMNs after BPAI

Ferroptosis is an iron-dependent, lipid peroxidation-driven form of regulated cell death that has recently been implicated in motor neuron vulnerability after severe nerve injury (Dixon et al., 2012; Stockwell et al., 2017). Accumulating evidence from BPAI, SCI, and peripheral nerve trauma models indicate that proximal SMNs exhibit key molecular hallmarks of ferroptosis, including iron dysregulation, glutathione depletion, GPX4 inactivation, and excessive lipid peroxidation.

Following root avulsion, axonal disconnection and mechanical stress disrupt mitochondrial homeostasis and intracellular iron handling, leading to reactive ROS accumulation and peroxidation of polyunsaturated fatty acids within neuronal membranes. Loss of glutathione-dependent antioxidant capacity-together with functional impairment of glutathione peroxidase 4 (GPX4)-renders SMNs highly susceptible to ferroptotic execution. These events occur early after injury and may persist, contributing to both neuronal loss and chronic neuropathic pain (Huang L. et al., 2022; Huang and Bai, 2024; Liu et al., 2025). Experimental evidence supports a causal role for ferroptosis in nerve injury-induced neurodegeneration. In rodent models of peripheral nerve injury and SCI, genetic ablation or pharmacological inhibition of GPX4 precipitates rapid motor neuron degeneration, whereas ferroptosis inhibitors such as ferrostatin-1, liproxstatin-1, iron chelators, or antioxidant supplementation significantly attenuate neuronal loss and improve functional recovery (Bai et al., 2023; Li W. et al., 2023; Liu et al., 2024). Recent studies further suggest that modulation of calcium-related signaling pathways can influence ferroptotic susceptibility in the injured spinal cord and alleviate neuropathic pain-associated behaviors, highlighting functional relevance beyond cell survival alone (Guo et al., 2021; Li L. et al., 2023).

### 4.5 Autophagy

Autophagy is a conserved lysosome-dependent degradation pathway essential for neuronal homeostasis, enabling the clearance of damaged organelles, misfolded proteins, and toxic aggregates (Mizushima and Levine, 2010). After BPAI, SMNs exhibit a robust but dysregulated autophagic response, which can exert either neuroprotective or neurotoxic effects depending on timing and flux integrity (Hara et al., 2006).

Early after axonal root avulsion, mitochondrial dysfunction, ATP depletion, reactive ROS accumulation, and intracellular Ca^2+^ overload activate the AMPK-ULK1-Beclin-1 signaling axis, triggering autophagosome formation. Increased expression of autophagy markers such as LC3-II and Beclin-1 has been detected in the spinal anterior horn within 24–48 h post-injury, consistent with an adaptive attempt to restore cellular homeostasis (Ribas et al., 2015). However, accumulating evidence indicates that autophagic flux is frequently impaired during the subacute and chronic phases after BPAI. Pathological upregulation of aldose reductase (AR) in the spinal ventral horn has been shown to suppress the SIRT1-AMPK-mTOR pathway, leading to defective autophagosome clearance, persistent oxidative stress, neuroinflammation, and progressive SMN loss. Genetic deletion of AR or pharmacological inhibition using epalrestat restores autophagic flux, reduces inflammatory cytokine production, preserves motor neuron survival, and significantly improves forelimb motor function in BPAI models (Zhong et al., 2022; Li et al., 2025).

Together, these findings support a dual-role model of autophagy in BPAI: transient activation of autophagy is initially protective, whereas sustained or dysregulated autophagy accelerates neurodegeneration. Therapeutic strategies aimed at normalizing autophagic flux, rather than globally enhancing or suppressing autophagy, may therefore represent an effective approach to preserve SMNs and promote functional recovery after BPAI.

Beyond its cell-autonomous role in neuronal homeostasis, autophagy dysregulation may sensitize SMNs to inflammatory and oxidative cues, providing a mechanistic bridge between intracellular stress responses and microenvironment-driven death amplification.

### 4.6 Microenvironment-driven death amplification: the immune-glia-neuron triangle after BPAI

Following brachial BPAI, SMN degeneration extends far beyond the initial mechanical insult, driven by a self-amplifying inflammatory microenvironment formed by interactions among glial cells, infiltrating immune cells, and neurons (Bellver-Landete et al., 2019; Zhong et al., 2021). SMN degeneration evolves through a temporally orchestrated inflammatory cascade. Within the acute phase (≤72 h), resident microglia rapidly acquire a pro-inflammatory phenotype and release TNF-α, IL-1β, IL-6, and ROS, initiating early apoptotic and pyroptotic signaling that accounts for the first wave of SMN loss. During the subacute phase (3–14 days), disruption of the blood-spinal cord barrier permits infiltration of monocyte-derived macrophages and T lymphocytes, which further amplify cytokine release, complement activation, and oxidative stress, thereby accelerating secondary motor neuron degeneration. In the chronic phase (>14 days), sustained astrocyte activation and excessive extracellular matrix deposition culminate in glial scar formation, chemically and physically isolating surviving SMNs, inhibiting axonal regeneration, and stabilizing a persistently neurotoxic microenvironment (Table 2) (Kigerl et al., 2009; Brennan et al., 2012; Komine and Yamanaka, 2015; Lacagnina et al., 2025; Saad et al., 2025).

**Table 2:** Microenvironmental drivers of secondary SMN degeneration after BPAI.

Phase after injury	Dominant cellular players	Major mediators	Impact on SMNs	Targetable pathways
Acute (≤72 h)	Microglia (M1-like)	Neuron–glia-immune network	Early apoptosis and pyroptosis	Minocycline; NF-κB inhibition; Hv1 blockade
Subacute (3–14 d)	Macrophages; T cells	Cytokines; Complement C5a	Necroptosis; Inflammatory amplification	C5aR antagonists; Cytokine neutralization
Chronic (>14 d)	Astrocytes; ECM-producing cells	CSPGs; CXCL10	Glial scarring; Regeneration failure	Anti-fibrotic strategies; CXCL10 inhibition
Cross-phase loops	Neuron-glia-immune network	TNF–NF-κB; IL-1β–NLRP3	Self-sustaining degeneration	Multi-axis immunomodulation

**Figure 3:**
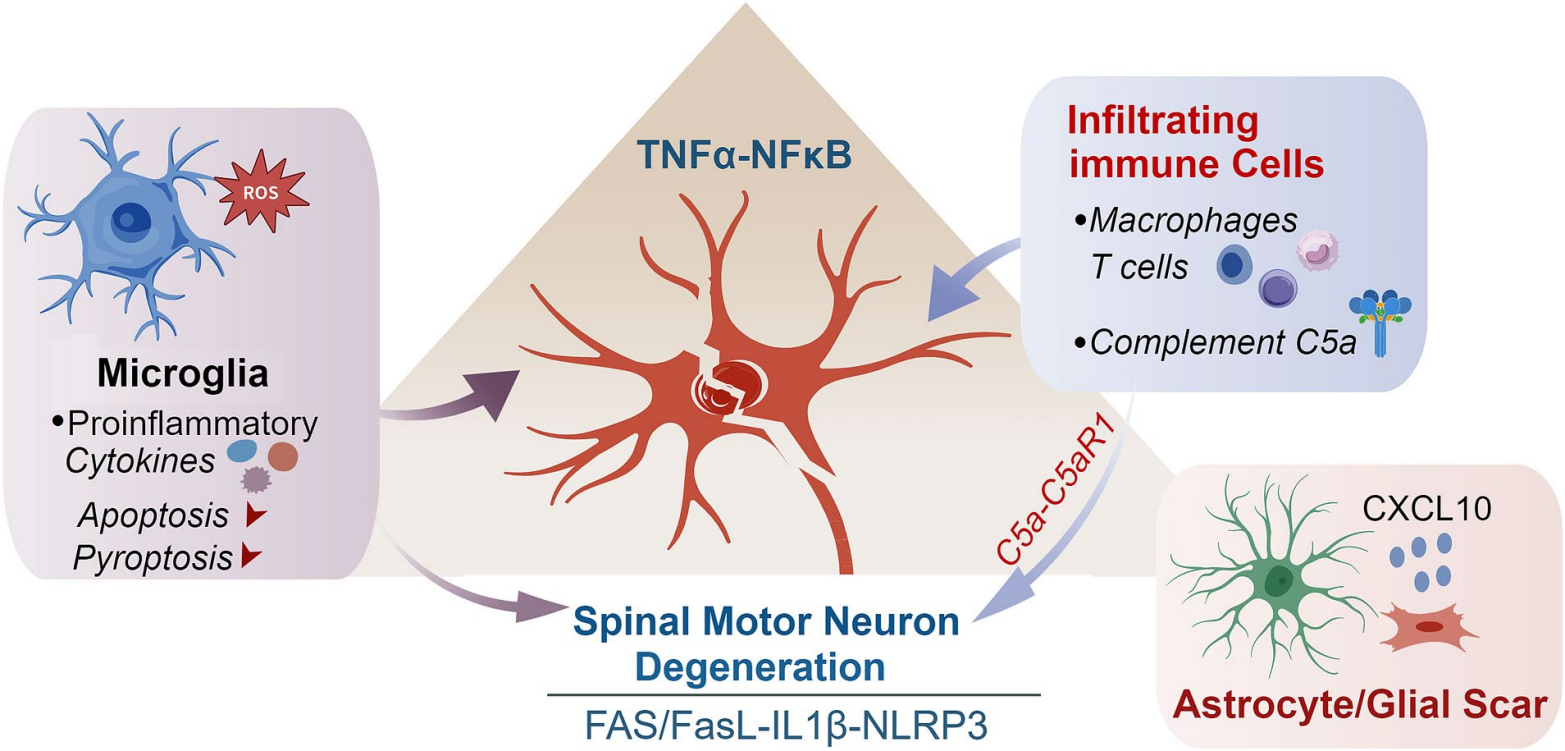
Microenvironment-driven death amplification: The immune-glia–neuron triangle after BPAI. This figure illustrates how interactions among neurons, glial cells, and infiltrating immune cells create a self-amplifying inflammatory microenvironment that accelerates SMN degeneration after BPAI. In the acute phase, activated microglia release pro-inflammatory cytokines (TNF-α, IL-1β, IL-6) and reactive oxygen species, initiating early apoptotic, necroptotic, and pyroptotic signaling. During the subacute phase, blood-spinal cord barrier disruption permits macrophage and T-cell infiltration, enhancing cytokine production, complement activation, and oxidative stress. In the chronic phase, sustained astrocyte activation and extracellular matrix deposition lead to glial scar formation, isolating surviving SMNs and inhibiting regeneration. Molecular feedback loops—including TNF-α-NF-κB-ROS signaling, IL-1β-NLRP3 inflammasome activation, complement C5a-C5aR1 signaling, and astrocyte-derived CXCL10—maintain this neurotoxic microenvironment, driving progressive and irreversible SMN loss after BPAI.

**Figure 4:**
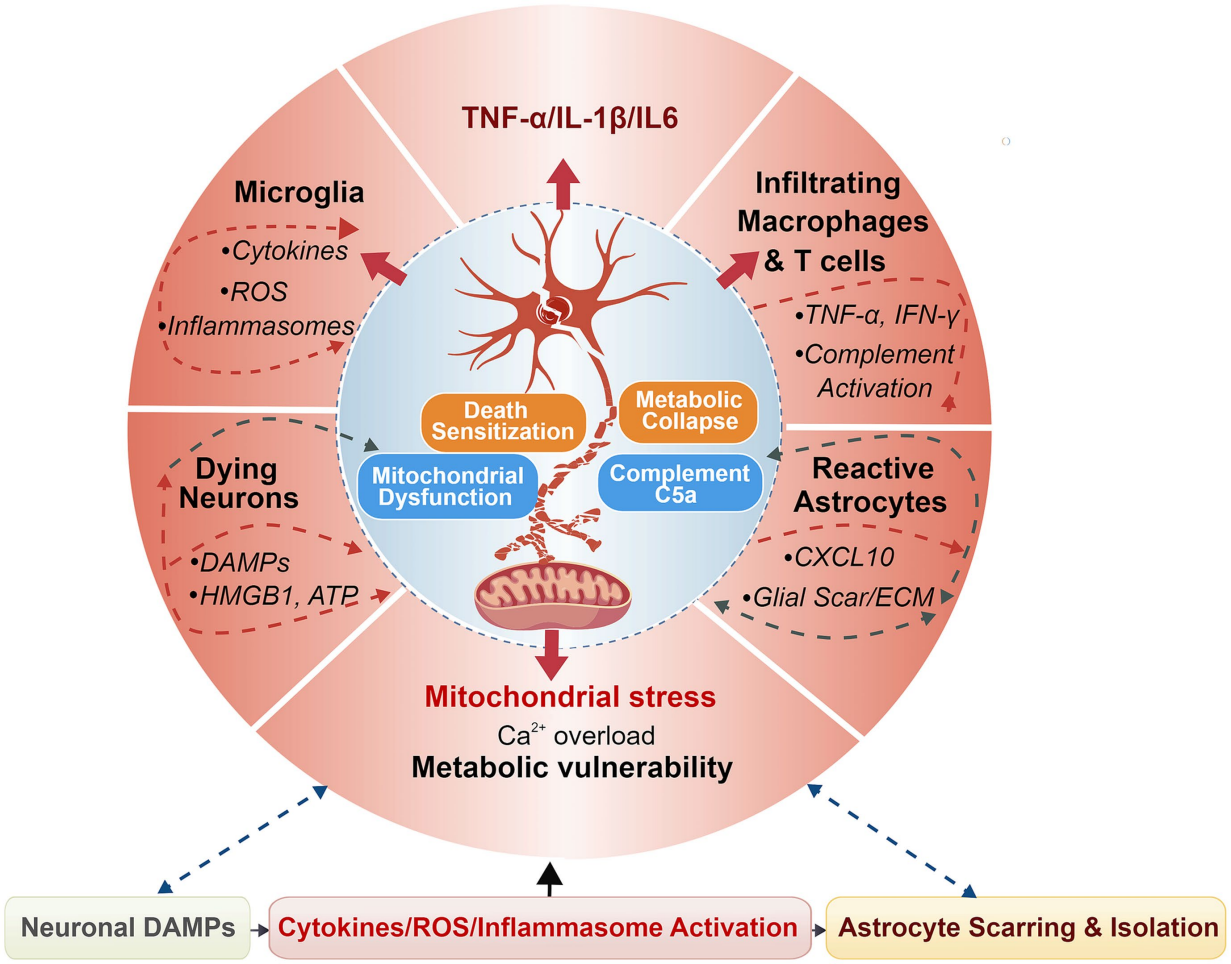
Inflammatory microenvironment-driven amplification of motor neuron death after BPAI. This schematic illustrates the interplay of inflammatory factors in the progression of motor neuron death following BPAI. At the center, a motor neuron is depicted, showing the effects of mitochondrial stress, Ca^2+^ overload, and metabolic vulnerability as a result of inflammation. Surrounding the neuron, key inflammatory stressors including pro-inflammatory cytokines (TNF-α, IL-1β, IL-6), reactive oxygen species (ROS), and complement activation from microglia, infiltrating macrophages, and T cells contribute to death sensitization, mitochondrial dysfunction, and metabolic collapse. Astrocyte-derived chemokines (CXCL10) and extracellular matrix (ECM) remodeling play a crucial role in isolating and preventing neuronal recovery through scarring. The outer rings highlight the cellular sources of these factors, emphasizing the reciprocal feedback loops between dying neurons, inflammatory cells, and neurodegenerative processes. Dashed arrows indicate feedback mechanisms that further amplify neurodegeneration, while solid arrows represent direct inflammatory signaling pathways.

At the molecular level, this immune-glia–neuron triangle is maintained by interconnected feedback loops, including TNF-α-NF-κB-ROS signaling, IL-1β-NLRP3 inflammasome activation, complement C5a-C5aR1-mediated necroptotic bias, and astrocyte-derived CXCL10-dependent immune recruitment (Figure 3). Together, these autonomous (neuronal) and non-autonomous (glial and immune) mechanisms create a self-sustaining inflammatory microenvironment that dictates irreversible proximal SMN loss after BPAI. These microenvironment-driven mechanisms underscore the therapeutic value of modulating immune-glia interactions to disrupt death-amplifying feedback loops and preserve spinal motor neuron viability after BPAI (Figure 4).

### 4.7 Single-cell transcriptomic insights into spinal motor neuron degeneration after BPAI

Recent advances in single-cell and single-nucleus RNA sequencing have provided valuable insights into the cellular heterogeneity and injury-induced transcriptional programs underlying spinal cord neurodegeneration. Although BPAI-specific single-cell datasets are currently limited, evidence from spinal cord injury models offers an important framework for understanding BPAI-associated SMN loss.

Single-cell analyses indicate that neurons in ventral spinal regions undergo dynamic and heterogeneous transcriptional changes after injury. Vulnerable neuronal populations exhibit downregulation of genes related to synaptic transmission and membrane excitability, alongside upregulation of stress-response, RNA-processing, autophagy-related, and metabolic pathways during chronic phases. These findings suggest that motor neurons may transition from an early regenerative or compensatory state toward maladaptive stress and catabolic programs, increasing susceptibility to programmed cell death (Fan et al., 2023).

In parallel, glial cells show robust and spatially heterogeneous activation. Microglia progressively acquire disease-associated phenotypes enriched for lipid metabolism, phagocytosis, and inflammatory signaling, while astrocytes and oligodendrocyte lineage cells display region-specific transcriptional remodeling. Notably, distal spinal segments below the injury develop a sustained degenerative microenvironment characterized by persistent microglial activation and altered neuronal composition, supporting the concept of long-range, non-cell-autonomous amplification of neurodegeneration (Floriddia et al., 2020).

Together, these single-cell transcriptomic findings support a network-based model in which BPAI-related SMN loss reflects the convergence of neuron-intrinsic stress responses and cell-extrinsic inflammatory and metabolic pressures imposed by a heterogeneous immune–glia microenvironment. Future BPAI-focused single-cell and spatial transcriptomic studies will be essential to delineate vulnerable SMN subpopulations and define stage-specific therapeutic windows.

## 5 Targeted intervention strategies: precision neuroprotection from molecular to clinical

**Figure 5:**
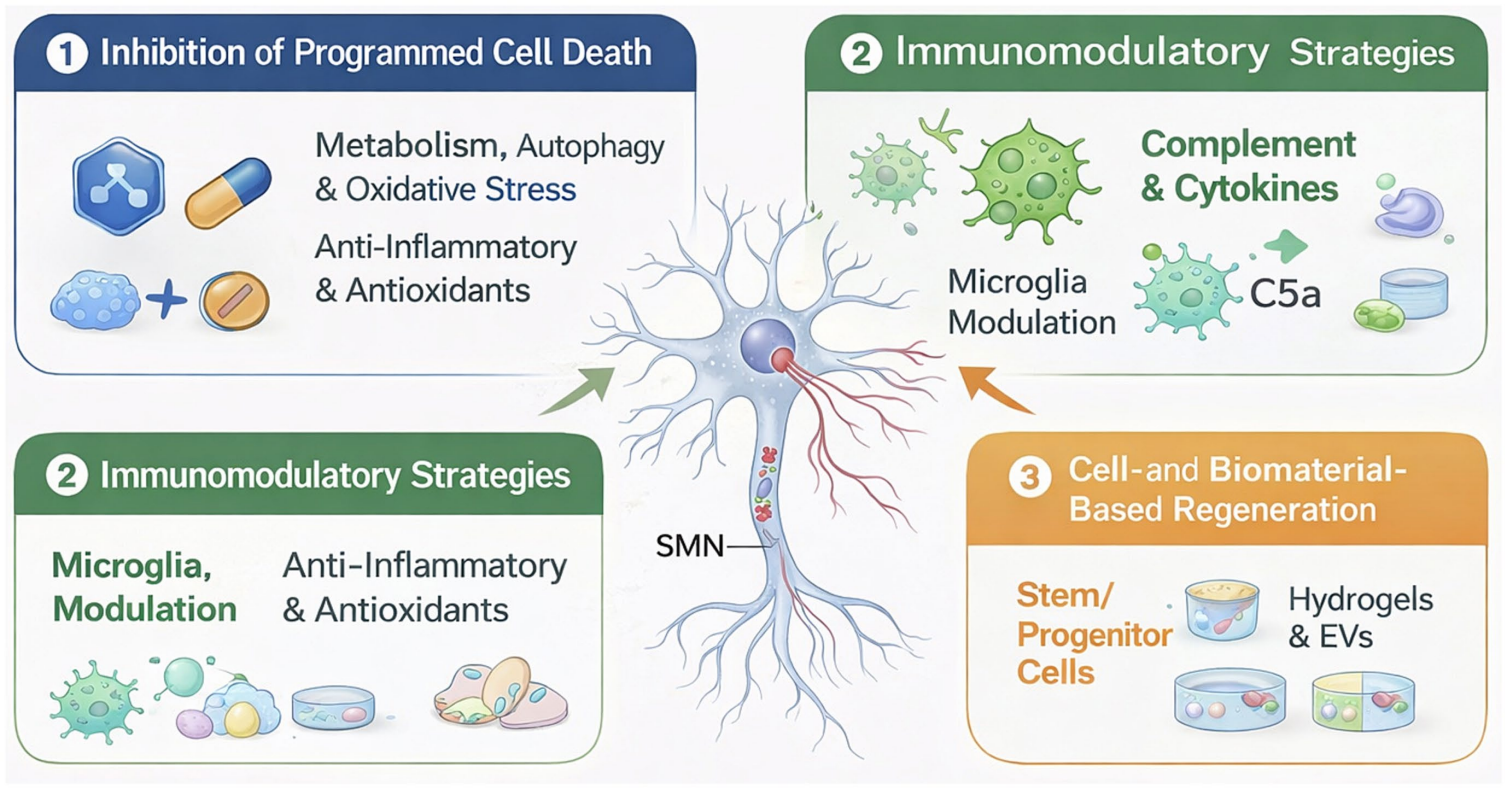
Precision neuroprotection strategies after BPAI. Schematic overview of targeted intervention strategies for SMN preservation and regeneration following BPAI. Root avulsion triggers convergent pathological processes, including metabolic stress, autophagy dysfunction, oxidative injury, and neuroinflammation, leading to activation of multiple programmed cell death pathways. Therapeutic approaches include inhibition of neuron-intrinsic death programs, immunomodulation of the immune-glia–neuron microenvironment, and cell- or biomaterial-based regenerative strategies. Together, these complementary interventions form an integrated precision neuroprotection framework that bridges molecular mechanisms with translational and clinical applications.

Targeted intervention strategies for BPAI aim to preserve SMN viability and promote functional recovery by integrating molecular, microenvironmental, and regenerative approaches. Precision neuroprotection focuses on inhibiting convergent programmed cell death pathways, modulating the inflammatory immune-glianeuron microenvironment, and applying advanced delivery systems to enhance therapeutic specificity and efficacy. Complementary cell- and biomaterial-based strategies further support neuronal survival, axonal regeneration, and circuit reconstruction (Table 3; Figure 5). Together, these multimodal interventions provide a translational framework bridging mechanistic insights with clinically actionable therapies for BPAI.

**Table 3:** Targeted intervention strategies for precision neuroprotection after BPAI.

Strategy category	Representative agents	Primary targets	Mechanistic effects	Preclinical outcomes
Metabolic/autophagy modulation	Epalrestat	Aldose reductase	Restores SIRT1-AMPK-mTOR; Normalizes autophagy	↑ SMN survival; ↑ Motor recovery
Anti-pyroptotic therapy	N-acetyl-L-glutamine (NAG)	NLRP3/caspase-1/GSDMD	Suppresses pyroptosis; Promotes remyelination	↓ Inflammation; ↑ Functional recovery
Antioxidant therapy	Quercetin	ROS; Glial activation	Reduces oxidative stress; Supports axonal growth	↑ MN survival; ↑ Regeneration
Anti-ferroptotic therapy	Ferrostatin-1	Lipid peroxidation	Blocks ferroptotic execution	↓ Neuronal loss; ↓ Pain
Advanced delivery	AAV-PHP.eB	GPX4; Neuroprotective genes	Efficient spinal gene transfer	↑ Neuronal resistance
EV-based therapy	MSC-EVs + hydrogels	Inflammation; Ferroptosis	Sustained immunomodulation	↑ Vascular repair; ↑ Locomotion
Cell-based regeneration	NSCs/NPCs	Trophic support; Circuit repair	Neurotrophic secretion; Synaptic support	↑ Reinnervation; ↑ Function

### 5.1 Inhibitors of the programmed death pathway

PCD-including apoptosis, pyroptosis, ferroptosis, and necroptosis-is a major driver of SMN loss following BPAI. Recent studies indicate that metabolic stress, autophagy failure, oxidative injury, and inflammatory amplification converge to lower neuronal survival thresholds, making pathway-specific inhibition a rational strategy for precision neuroprotection.

#### 5.1.1 Metabolic and autophagy-targeted interventions

Metabolic dysregulation critically contributes to SMN vulnerability after root avulsion. Genetic deletion or pharmacologic inhibition of AR restores SIRT1-AMPK-mTOR signaling, normalizes autophagic flux, reduces lipid peroxidation and neuroinflammation, and significantly preserves SMN survival. The clinically approved AR inhibitor epalrestat reproduces these effects in rodent BPAI models, highlighting AR as a metabolically driven amplifier of neuronal death (Zhong et al., 2022).

#### 5.1.2 Anti-inflammatory and antioxidant small molecules

Small molecules targeting oxidative stress and inflammasome activation provide complementary neuroprotection. N-acetyl-L-glutamine (NAG) enhances motor recovery, suppresses NLRP3/caspase-1/GSDMD-mediated pyroptosis, promotes remyelination, and improves SMN survival in nerve injury models (Wu L. et al., 2023). Quercetin, a pleiotropic flavonoid, attenuates glial activation, reduces ROS accumulation, and supports axonal regeneration and motor neuron preservation across SCI and peripheral nerve injury paradigms (Fideles et al., 2023). Together, these agents interrupt a death-amplification loop linking metabolic stress, autophagy disruption, ROS accumulation, and inflammatory PCD.

#### 5.1.3 Advanced delivery strategies

Therapeutic efficacy is further enhanced by spinal-targeted delivery systems. AAV-PHP.eB enables efficient CNS gene transfer of anti-ferroptotic factors such as GPX4 in rodents (Chan et al., 2017). Local delivery of ferroptosis inhibitors-most notably ferrostatin-1-via ROS-responsive hydrogels maintains effective drug concentrations in oxidative microenvironments and reduces lipid peroxidation-driven neuronal loss (Skouta et al., 2014; Hua et al., 2024). In parallel, dexamethasone-loaded mesenchymal stem cell-derived extracellular vesicles embedded in injectable hydrogels suppress inflammation, modulate ferroptosis and pyroptosis, and improve functional recovery in spinal injury models (Cao et al., 2025).

#### 5.1.4 Translational perspective

Collectively, these studies support a precision neuroprotective framework in which clinically accessible small molecules (e.g., epalrestat, NAG, quercetin, ferrostatin-1), combined with advanced spinal delivery platforms, can simultaneously modulate multiple convergent death pathways. Prioritizing agents that penetrate the spinal cord, target overlapping PCD mechanisms, and demonstrate functional benefit in large-animal models will be essential for translating programmed death inhibition into effective clinical therapies for BPAI.

### 5.2 Immunomodulatory and microenvironment-targeted strategies

Beyond neuron-intrinsic death pathways, a pathological immune-glia–neuron microenvironment critically amplifies SMN loss after BPAI. Activated glial cells, infiltrating immune populations, and inflammatory mediators form self-reinforcing feedback loops that propagate apoptosis, necroptosis, and pyroptosis. Targeting this microenvironment therefore represents a complementary and clinically relevant neuroprotective strategy.

#### 5.2.1 Microglial modulation

Microglia rapidly adopt a pro-inflammatory (M1-like) phenotype after BPAI, releasing TNF-α, IL-1β, IL-6, and ROS that exacerbate SMN death. Pharmacological agents such as minocycline effectively suppress microglial activation, reduce cytokine production, and limit secondary motor neuron loss in SCI and peripheral nerve injury models (Stirling et al., 2004; Garrido-Mesa et al., 2013). In parallel, IL-4-mimetic strategies promote M2-like polarization, enhancing anti-inflammatory signaling, trophic support, and neuronal survival (Daines et al., 2021). Gene-based approaches targeting microglial signaling pathways-including NF-κB inhibition or Hv1 proton channel knockdown-further attenuate oxidative stress and inflammasome activation, preserving SMN integrity (Li et al., 2021).

#### 5.2.2 Complement and cytokine pathway inhibition

Complement activation, particularly through the C5a-C5aR1 axis, amplifies neuroinflammation and shifts neuronal death toward necroptosis and pyroptosis. Pharmacological C5aR antagonists reduce microglial recruitment and complement-driven neuronal injury (Brennan et al., 2012; Ge et al., 2026). Likewise, neutralization of key cytokines-TNF-α, IL-1β, and IL-6-using antibodies or decoy receptors dampens inflammatory signaling, limits glial reactivity, and mitigates SMN degeneration.

#### 5.2.3 Restoration of blood-spinal cord barrier integrity

Disruption of the blood–spinal cord barrier (BSCB) after BPAI permits infiltration of peripheral immune cells, intensifying local inflammation. Matrix metalloproteinase inhibitors preserve extracellular matrix integrity, while endothelial-protective agents (e.g., angiopoietin-1 mimetics) stabilize tight junctions and reduce immune cell extravasation (Venkat et al., 2018). In parallel, hydrogel-based biomaterials delivering anti-inflammatory or neurotrophic factors locally to the spinal cord limit immune infiltration while supporting neuronal survival (Cao et al., 2025).

#### 5.2.4 Combined microenvironmental approaches

Emerging evidence supports synergistic strategies that simultaneously modulate multiple microenvironmental axes. Dexamethasone-loaded MSC-derived extracellular vesicles (EVs) embedded in ROS-responsive hydrogels suppress glial activation, reduce pyroptosis, improve microvascular perfusion, and enhance SMN survival (Cao et al., 2025). In addition, CD24Fc and engineered stem cell-based therapies reprogram innate immune responses, attenuating both acute inflammation and chronic glial scarring (Liu and Zheng, 2023).

#### 5.2.5 Translational perspective

Immunomodulatory strategies complement neuron-intrinsic neuroprotection by disrupting self-amplifying inflammatory circuits within the injured spinal cord. For successful clinical translation, interventions must be deployed within the early post-injury window, target multiple inflammatory and complement pathways, and preserve a permissive spinal microenvironment to support subsequent axonal regeneration and functional reconstruction.

### 5.3 Cell- and biomaterial-based regenerative strategies

While inhibition of PCD and immunomodulation preserve proximal SMNs, durable functional recovery after BPAI ultimately requires structural, synaptic, and circuit-level reconstruction. Cell- and biomaterial-based strategies provide integrated platforms for neuroprotection, axonal guidance, and reinnervation.

#### 5.3.1 Neural stem cells and progenitor transplantation

NSC/NPC transplantation into the spinal cord or avulsed root supplies both paracrine neurotrophic support and potential neuronal replacement. NSCs secrete GDNF, BDNF, and NT-3, promoting SMN survival, axonal sprouting, remyelination, and synaptic maintenance (Kadoya et al., 2016; Assinck et al., 2017). Genetically engineered NSCs expressing anti-apoptotic or anti-ferroptotic molecules (e.g., Bcl-xL, GPX4) further enhance motor neuron preservation in preclinical SCI and root avulsion models. Importantly, transplantation during the early post-injury window (1–2 weeks) maximizes graft integration and limits chronic denervation (Chen et al., 2021; Wu Y. et al., 2023).

#### 5.3.2 Extracellular vesicle-based therapies

Mesenchymal stem cell (MSC)- or NSC-derived EVs deliver microRNAs, trophic factors, and immunomodulatory cargo without the risks associated with cell engraftment. Hydrogel-encapsulated or 3D-culture-derived EVs enable sustained local release, suppress microglial activation, reduce pyroptosis and oxidative stress, and improve axonal regeneration. EV-based therapies have consistently demonstrated improvements in locomotor recovery, neuromuscular junction stability, and neuronal survival in SCI and BPAI-relevant models (Silva et al., 2021; Poongodi et al., 2025).

#### 5.3.3 Neural organoid and mini-spinal cord constructs

Spinal cord organoids pre-patterned toward a ventral motor neuron identity represent an emerging approach for *de novo* circuit reconstruction. When combined with bioengineered scaffolds, organoids can survive transplantation, establish synaptic connectivity with host neurons, and potentially bridge neuronal loss caused by avulsion. Although still experimental, this strategy offers a scalable platform for future reconstructive neurology (Olmsted and Paluh, 2021; Huang et al., 2024).

#### 5.3.4 Biomaterial scaffolds and hydrogels

Biomaterials provide structural and biochemical cues essential for regeneration. Fibrin, collagen, and decellularized nerve matrices support axonal alignment and Schwann cell migration, while ROS-responsive or growth factor-loaded hydrogels enable localized, sustained delivery of trophic and anti-degenerative agents. Hybrid systems integrating cells, EVs, and hydrogels achieve multimodal neuroprotection by simultaneously supporting SMNs, modulating inflammation, and guiding axonal regrowth (Guijarro-Belmar et al., 2022; Cao et al., 2025).

#### 5.3.5 Translational considerations

Clinically viable regenerative strategies must preserve host SMNs, integrate with surviving neural circuits, and demonstrate long-term safety and functional benefit. The convergence of cell therapy, EV delivery, organoid engineering, and smart biomaterials, particularly when combined with molecular and immunomodulatory interventions, represents a precision regenerative framework for restoring motor function after BPAI.

Despite encouraging progress in preclinical models, the translation of neuroprotective strategies for BPAI into clinical practice faces several substantial challenges. First, the therapeutic time window is narrow, as SMA degeneration progresses rapidly after avulsion, making early diagnosis and timely intervention critical yet difficult in real-world settings. Second, effective delivery of pharmacological agents, biologics, or gene therapies across the blood-spinal cord barrier remains a major obstacle, particularly for achieving sufficient concentration within the ventral horn while minimizing systemic toxicity. Third, the extensive redundancy and crosstalk among programmed cell death pathways raise concerns that targeting a single mechanism may be insufficient, necessitating combination or stage-specific interventions that increase complexity and safety considerations. In addition, many promising approaches rely on rodent models, which may not fully recapitulate the anatomical scale, biomechanical forces, and chronic inflammatory milieu observed in human BPAI. Finally, long-term safety, off-target effects, and regulatory feasibility of advanced therapies-including biomaterial-based delivery systems and cell-based interventions-require rigorous evaluation. Addressing these challenges will be essential for bridging the gap between mechanistic insights and effective clinical neuroprotection after BPAI.

## 6 Conclusion

BPAI represents a uniquely severe form of neurotrauma in which proximal SMN degeneration, distal target denervation, and maladaptive microenvironmental remodeling converge to fundamentally constrain functional recovery. Accumulating evidence indicates that SMN loss after BPAI is not driven by a single pathological process but rather arises from the coordinated activation of multiple programmed cell death (PCD) pathways, including apoptosis, necroptosis, pyroptosis, ferroptosis, and autophagy dysregulation. Importantly, the strength and nature of evidence supporting these pathways are not uniform. Apoptosis, neuroinflammation-associated neuronal injury, and impaired autophagic flux are supported by direct experimental evidence from BPAI and root avulsion models, whereas the involvement of necroptosis, pyroptosis, ferroptosis, and epitranscriptomic regulation is currently inferred largely from spinal cord injury and peripheral nerve injury studies that share key pathological features with BPAI, such as axonal transection, metabolic stress, and sustained inflammation.

Available data further suggest a stage-dependent yet largely parallel activation pattern of PCD pathways. Apoptotic signaling appears to be most prominent during the acute post-injury phase, while necroptotic, pyroptotic, ferroptotic, and autophagy-related mechanisms become increasingly relevant during subacute and chronic stages. Rather than operating as a strictly sequential cascade, these pathways exhibit extensive crosstalk and mutual reinforcement within a dynamic injury network. This network is tightly coupled to a tri-phasic immune-glia–neuron microenvironment, in which microglial activation, astrocytic reactivity, peripheral immune cell infiltration, oxidative stress, and metabolic dysregulation form self-perpetuating death-amplification loops that extend neuronal degeneration well beyond the initial mechanical insult.

This evolving pathological network establishes a critical biological barrier that fundamentally limits the efficacy of conventional surgical reconstruction alone. Even when anatomical continuity is restored, persistent neuroinflammation, redox imbalance, and bioenergetic failure within the spinal cord compromise durable motor unit preservation and functional reinnervation. Recognition of this biology reframes BPAI as a combined neurodegenerative and regenerative disorder, rather than a purely peripheral nerve injury.

Emerging mechanisms such as the METTL14-EEF1A2-m^6^A axis and the AR-SIRT1-AMPK-mTOR pathway likely represent conserved stress-response programs observed across diverse forms of neural injury, which may be particularly pronounced in BPAI due to the severity of axonal disconnection and energy collapse. Together with growing single-cell transcriptomic evidence revealing cell-type-specific vulnerability and microenvironmental heterogeneity, these findings underscore the importance of integrated, system-level approaches to understanding SMN degeneration.

Recent preclinical advances highlight the feasibility of precision neuroprotective strategies, in which pathway-specific inhibition of PCD, targeted immunomodulation, and cell- or biomaterial-based regenerative platforms act in concert to preserve SMNs, suppress inflammatory amplification, and support axonal regeneration. Importantly, therapeutic timing emerges as a decisive determinant of efficacy, as intervention during the early post-injury “golden window” is critical to interrupt death-amplification loops before irreversible circuit collapse occurs. Nonetheless, significant challenges remain for clinical translation, including optimal timing of intervention, effective delivery across the blood-spinal cord barrier, pathway redundancy, long-term safety, and the limited predictive value of current preclinical models.

Looking forward, the most promising translational trajectory lies in integrated, multimodal therapeutic paradigms that simultaneously address intrinsic neuronal vulnerability and extrinsic microenvironmental toxicity. Key future priorities include defining causal hierarchies among PCD pathways, identifying vulnerable versus resilient SMN subpopulations using single-cell and spatial transcriptomic approaches, and delineating stage-specific therapeutic windows. Progress toward large-animal validation and carefully designed clinical trials will be essential for shifting BPAI management from delayed anatomical repair toward true biological and functional restoration. Ultimately, decoding and modulating the reciprocal interplay between SMN death programs and the neuroimmune microenvironment will form the foundation of next-generation precision neuroregenerative strategies for severe peripheral nerve injuries.
